# A Dangerous Region Generation Method for Computer-Assisted Pelvic Bone Tumor Resection Surgery: A Retrospective Study

**DOI:** 10.3390/jcm15031034

**Published:** 2026-01-28

**Authors:** Daming Pang, Zhuoyu Li, Yang Sun, Weifeng Liu, Yu Zhang, Qing Zhang

**Affiliations:** 1 Department of Orthopaedic Oncology, Affiliated Beijing Jishuitan Hospital, Capital Medical University, Beijing 100035, China; 2 School of Astronautics, Beihang University, Beijing 100191, China

**Keywords:** pelvic bone tumor, computer-assisted bone tumor resection surgery, dangerous region generation, 3D image resampling

## Abstract

**Background:** Achieving adequate margins in pelvic bone tumor resection remains difficult, as conventional navigation provides no direct three-dimensional margin feedback. We proposed an innovative dangerous region generation method based on 3D image resampling and anisotropic distance transform, integrated with computer-assisted navigation, to enhance surgical margin accuracy. This study aimed to evaluate its oncological safety, functional outcomes, and perioperative efficacy in pelvic tumor surgery. **Methods:** The study was conducted on 19 patients (8 males, 11 females) with primary pelvic bone tumors between May 2018 and June 2024. The age range was 19 to 66 years (mean age: 62.67 years). Histological diagnoses included chondrosarcoma (*n* = 6), giant cell tumor (*n* = 4), osteosarcoma (*n* = 1), chordoma (*n* = 2), Ewing sarcoma (*n* = 3), spindle cell sarcoma (*n* = 1), chondromyxoid fibroma (*n* = 1), and peripheral nerve sheath tumor (*n* = 1). The feasibility of the dangerous region generation method for computer-assisted pelvic tumor resection surgery was assessed by general results, oncological and functional results. **Results:** All patients successfully underwent surgery with a mean operative time of 252 min and average intraoperative blood loss of 1358 mL. The mean hospital stay was 22 days, and all patients completed follow-up (mean, 37 months). Two patients developed postoperative wound complications, which resolved after debridement. Adequate surgical margins were achieved in all cases. The 5-year overall survival rate was 75.6%, increasing to 80.0% among patients with wide-margin resections. At the final follow-up, the mean MSTS score among 16 limb-salvage patients was 26.6, corresponding to an average functional recovery of 88.5%. Most patients exhibited a normal gait and were able to ambulate without assistive devices. **Conclusions:** This dangerous region generation method, when combined with computer-assisted techniques for pelvic bone tumor resection, is feasible and can achieve favorable clinical outcomes.

## 1. Introduction

The precise resection of bone tumors is a critical determinant of both oncological safety and postoperative functional preservation [1]. Pelvic bone tumors account for a minority (3–4%) of primary bone tumors. Their location within the complex pelvic anatomy renders complete surgical resection challenging, often leading to poor clinical outcomes. Achieving negative surgical margins remains the cornerstone of tumor resection, as inadequate margins or microscopic residual disease substantially increase the risk of local recurrence and adversely affect long-term survival [2]. Conversely, pursuing wider safety margins may result in unnecessary over-resection, leading to the loss of substantial amounts of uninvolved bone and adjacent soft tissue. This challenge is particularly pronounced in anatomically complex regions such as the pelvis or near major joints, where excessive resection can compromise limb function and significantly diminish patients’ quality of life [3,4].

Balancing complete tumor removal with maximal preservation of native structures underscores the clinical importance of accurate surgical planning and execution. Precisely defined osteotomy planes not only reduce the risk of residual disease but also facilitate optimal biomechanical reconstruction, thereby enhancing postoperative mobility and the long-term durability of prosthetic or biological reconstructions. With the growing adoption of computer-assisted navigation systems, preoperative planning has assumed even greater significance [5]. In current practice, orthopedic surgeons typically design osteotomy planes outside the tumor with a presumed safety margin, which are then used intraoperatively for navigation-guided guidance [6,7,8].

However, without direct feedback on the actual three-dimensional distance between planned osteotomy planes and tumor margins, surgeons cannot guarantee that the true margin consistently meets or exceeds the intended safety threshold [6,9,10,11]. If the actual distance falls short, the risk of recurrence and metastasis rises; conversely, if the distance greatly exceeds the planned margin, healthy bone is unnecessarily sacrificed. Both scenarios negatively impact postoperative function.

To address these challenges, Paul et al. developed haptic feedback devices to enhance the spatial accuracy of osteotomy plane placement [11]. While surgeons trained with this system could achieve target plane positions, the approach lacked intuitive clinical applicability and proved impractical for routine use. Ren et al. [12] proposed direct morphological dilation of the tumor region to incorporate a safety margin, yet anisotropic voxel spacing in medical imaging often caused substantial deviation from the true anatomical boundaries. In our previous work, we introduced a dangerous region generation method that constructs a patient-specific region encompassing the tumor and its surrounding safety margins [2]. This technique converts both the bone and tumor regions into three-dimensional point sets and iteratively accumulates bone points located within the safety margin of each tumor point to generate the final dangerous region. Compared with morphological dilation, this point-based approach improved boundary accuracy [12]. Clinically, the generated region can be imported into surgical navigation or planning systems, enabling surgeons to place osteotomy planes outside the tumor while maintaining adequate margins. However, this iterative method suffers from lower computational efficiency and slight accuracy loss due to information reduction during voxel-to-point conversion.

To overcome these limitations, we further developed a dangerous region generation method based on 3D image resampling and anisotropic distance transform. This approach rapidly produces patient-specific three-dimensional dangerous regions encompassing the tumor and adjacent tissues within the safety margin, achieving substantially higher spatial accuracy than conventional methods. When integrated with computer-assisted navigation, this technique facilitates more precise tumor resections.

In this study, we retrospectively analyzed cases of pelvic bone tumor resections performed at our institution using the proposed dangerous region generation method combined with computer-assisted surgical techniques. We evaluated oncological outcomes, functional recovery, and perioperative parameters to assess the safety and clinical efficacy of this integrated approach in pelvic tumor surgery.

## 2. Materials and Methods

### 2.1. Patients’ Involvement

**Figure 1:**
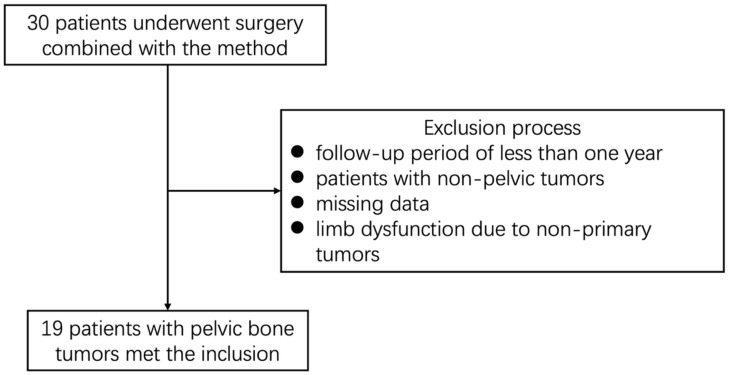
Flowchart of the study group.

This study was conducted as a retrospective analysis and was approved by the Medical Ethics Committee of Beijing Jishuitan Hospital. We retrospectively reviewed the data of patients with pelvic bone tumors who underwent computer-assisted surgery combined with the dangerous region generation method at our institution between May 2018 and June 2024. The inclusion and exclusion criteria are shown in the flowchart (Figure 1).

A total of 19 patients with primary pelvic bone tumors were ultimately included in the study cohort, comprising 11 females and 8 males, with an age range of 19 to 66 years (mean age: 62.67 years). Histological diagnoses included chondrosarcoma (*n* = 6), giant cell tumor (*n* = 4), osteosarcoma (*n* = 1), chordoma (*n* = 2), Ewing sarcoma (*n* = 3), spindle cell sarcoma (*n* = 1), chondromyxoid fibroma (*n* = 1), and peripheral nerve sheath tumor (*n* = 1).

The pelvic bone resection as defined by Enneking and Dunham [13] were typed I in 4 patients, typed II in 2 patients, type III in 5 patients, type IV in 2 patients, type II + III in 4 patients and type I + II in 2 patients. According to the Enneking surgical staging system [14], 5 patients were classified as Stage III, 6 as Stage IB, and 8 as Stage IIB (Table 1).

### 2.2. Dangerous Region Generation Method

For each surgical plan, CT and MRI data within 4 weeks prior to surgery were used to reconstruct patient-specific three-dimensional (3D) bone and tumor models. Tumor and bone masks were annotated in Mimics (version 16.0, Materialise n.v., Leuven, Belgium) by an orthopedic oncologist with more than 3 years of experience and subsequently verified by a senior orthopedic oncologist with over 10 years of experience. These annotated masks served as the basis for generating individualized dangerous regions for each patient.

**Figure 2:**
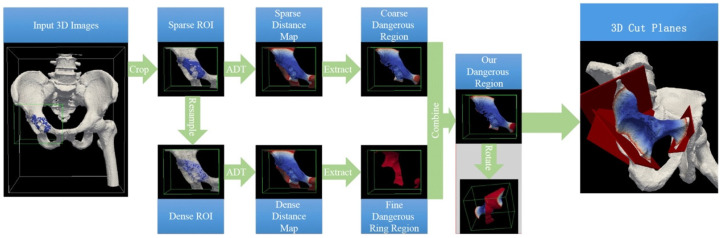
Flowchart of our proposed dangerous region generation method [15]. In this figure, the green box marks the bounding area of the extracted ROIs, while ADT denotes the three-dimensional anisotropic distance transform approach. The surface coloration of the 3D models in the lower three rows represents the Euclidean distance from each point to the tumor region, with colors shifting toward red as the position approaches the tumor’s safe margin. It should be emphasized that our method is carried out on 3D images; however, for enhanced visualization, the workflow is demonstrated using 3D models reconstructed from these images.

The dangerous region generation was performed using a combination of Mimics (for image segmentation and 3D model reconstruction) and MATLAB (version R2019b, MathWorks Inc., Natick, MA, USA; for algorithmic processing and region generation). The overall procedure is outlined as follows (Figure 2):

(1) Cropping Regions of Interest (ROIs): The process begins by cropping regions of interest from both the 3D bone image and the 3D refined tumor image. Bounding boxes are defined around these areas to ensure the cropped regions are large enough to capture both the bone and tumor, along with the necessary surrounding tissues. Specifically, the bounding boxes for voxels undergoing anisotropic distance transform (ADT) were extended by approximately 20.0 mm beyond the actual safe margin plus the bone tumor region.

(2) 3D Image Resampling: After cropping, each ROI is resampled to achieve higher spatial resolution, producing more accurate representations of the tumor and adjacent tissues. All CT volumes were resampled to isotropic 1 mm spacing before annotation to maintain geometric consistency across cases. The resampling process enhances the precision of the subsequently generated dangerous region.

(3) Applying 3D Anisotropic Distance Transform (ADT): The 3D anisotropic distance transform is applied to both the original and resampled ROIs to compute the distance of each voxel from the tumor or bone surface. This step generates two distance maps: a sparse map for the original ROI and a dense map for the resampled ROI. The ADT input parameters include the spatial resolutions along each dimension of the input volume. For the original ROI, these correspond to the resolutions of the original DICOM images, whereas for the resampled ROI, they are adjusted according to the resampling ratio.

(4) Generating Coarse and Fine Dangerous Regions: Using the distance maps, the method generates two types of dangerous regions: a coarse dangerous region from the sparse distance map, and a fine dangerous ring region from the dense distance map. These regions represent the areas surrounding the tumor that should be removed to ensure a safe margin.

(5) Combining the Coarse and Fine Dangerous Regions: The final dangerous region is created by combining the coarse and fine dangerous regions. This step refines the representation of the dangerous area, balancing both accuracy and computational efficiency.

(6) Final Dangerous Region: The combined 3D dangerous region is represented as a point set and reconstructed into a closed 3D surface using the Delaunay triangulation method (inline function of MATLAB). In this study, the shrink factor S for triangulation was set to 1. The final model provides surgeons with a precise and intuitive reference for verifying that preplanned osteotomy planes maintain adequate margins during bone tumor resection.

By integrating 3D image resampling with anisotropic distance transformation, the proposed method achieves high precision, rapid computation, and strong clinical adaptability. On our testing platform (Intel Core i7-11700K CPU, 128 GB RAM, NVIDIA TITAN Xp GPU), the complete generation process for a typical 3D case required approximately 26.8 s, demonstrating its practicality for clinical preoperative planning.

The flowchart diagram and technical description can be found in detail in our technical report [15].

### 2.3. Surgical Procedures

**Figure 3:**
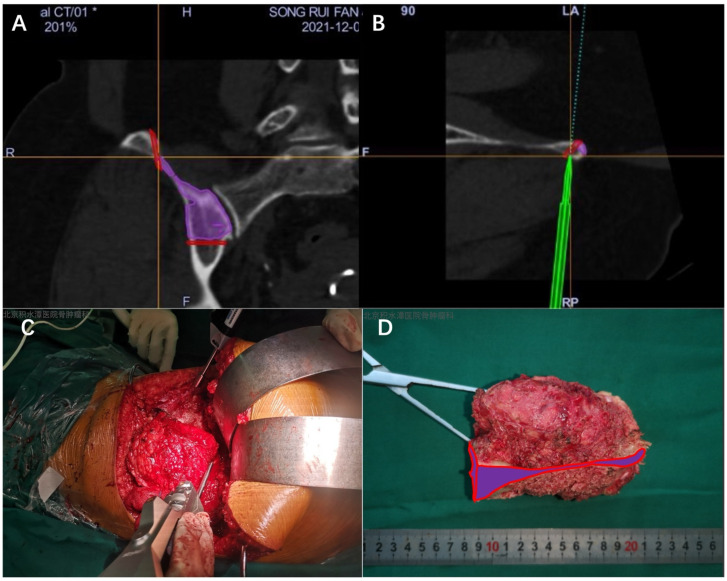
(**A**,**B**) The osteotomy trajectory was marked by the computer navigation guide according to the osteotomy plane designed before operation. (**C**) Osteotomy was performed under the guidance of a navigation guide; (**D**) Wide resection specimen of pelvic tumor. The purple area in the figure indicates the osteotomy plane.

All tumor resections were performed by experienced orthopedic oncologists. The surgical planning for pelvic tumors commonly follows the Enneking classification, which divides the pelvis into four zones: Zone I (the iliac wing), Zone II (the acetabulum and periacetabular region), Zone III (the pubic and ischial bones), and Zone IV (the sacrum and sacroiliac junction). Tumors often involve multiple adjacent zones, requiring combined resections (e.g., I + II or II + III), which further increase surgical complexity. Surgeons performing the computer-assisted navigation procedures had received formal, standardized training. After induction of anesthesia, the tumor site was exposed using a standard surgical approach with careful soft tissue dissection. Intraoperatively, tracking markers were placed on nearby bony landmarks adjacent to the incision. A 3D scan was then obtained using a C-arm imaging system (ARCADIS Orbic, Siemens, Germany), and the images were imported into the OrthoMap 3D navigation software (version 2.0-22, Stryker, Leibinger GmbH & Co. KG, Freiburg, Germany). These intraoperative scans were fused and registered with the preoperative CT and MRI data. Guided by the preoperatively planned dangerous region, the osteotomy boundaries were identified using a navigation probe, and the bone resection was carried out accordingly with high precision (Figure 3).

(1) Limb-Salvage Surgical Procedure:

Positioning and Surgical Approach: Patients were placed in the lateral decubitus position under general anesthesia. The surgical exposure was achieved through an ilioinguinal approach. The incision extended posterolaterally to the inferior border of the sacroiliac joint and anteromedially to the pubic symphysis, following the pubic ramus toward the ischial tuberosity. This allowed for complete exposure of the outer table of the ilium and the hip joint capsule, which was incised to dislocate the femoral head. On the medial side, the pubic symphysis was exposed, and the muscular attachments to the pubic and ischial rami, along with the sacrotuberous and sacrospinous ligaments, were detached. Abdominal muscle insertions on the iliac and pubic bones were released to expose the common, internal, and external iliac vessels and the femoral vessels. The femoral nerve was isolated and preserved. The iliopsoas muscle was dissected or divided when necessary to allow visualization of the inner ilium and anterior sacroiliac joint.

Iliac Osteotomy: At least 1 cm of soft tissue coverage over the tumor was preserved. Under navigation guidance, bone dissection was confirmed to be outside the planned osteotomy margins. Osteotomy trajectories were marked on both the inner and outer tables of the ilium based on the preoperative plan. After confirming consistency between the inner and outer osteotomy lines, iliac cuts were performed using a microsagittal saw or burr.

Partial Acetabular Osteotomy: The joint capsule was exposed, and the femoral head was dislocated to access the acetabulum. The osteotomy direction and extent were guided by the navigation system according to the preoperative plan. A navigation probe was used on the anterior aspect to confirm alignment of internal and external acetabular osteotomy lines. The acetabular resection was then completed using a microsagittal saw or ultrasonic bone scalpel, preserving part of the native acetabulum.

Low Sacroiliac Osteotomy with Sacral Nerve Preservation: The navigation system was used to identify and mark the posterior surface of the sacrum for osteotomy. The cut was performed with a burr, curette, and osteotome along the guided direction while carefully preserving the sacral nerves and adjacent vasculature.

Verification of Osteotomy Boundaries: After tumor removal, a navigation probe was used to compare the actual osteotomy surface with the preoperative plan point-by-point, ensuring the resection margins were not smaller than the planned boundaries.

(2) Amputation Procedure:

Positioning and Surgical Approach: The patient was placed in the lateral decubitus position under general anesthesia. A combined ilioinguinal approach was utilized. The incision extended anteromedially toward the pubic symphysis, following the pubic ramus to the ischial tuberosity, and was merged with a lateral incision running from the anterior superior iliac spine through the greater trochanter, aligned along the gluteal skin crease to join the medial limb of the incision.

Vascular and Nerve Management: The external iliac artery and vein were exposed on the anterior side, ligated, and transected. The femoral nerve was isolated and divided. The sciatic nerve was identified either medially or laterally to the inferior border of the sacroiliac joint, ligated, and then transected.

Osteotomy: A guiding saw was used to transect the pubic symphysis. Starting from the iliac wing, the insertions of the gluteus maximus and tensor fasciae latae were detached. The myocutaneous flap was elevated proximally to expose the outer table of the ilium down to the inferior sacroiliac joint. Osteotomy of the ilium and partial sacrum was performed similarly to the limb-salvage procedure. Under navigation guidance, osteotomy lines were marked and verified to ensure accurate alignment between the inner and outer surfaces of the ilium and the anterior and posterior sacral surfaces. A microsagittal saw or high-speed burr was used to complete the bone resection.

Ligamentous Division and Limb Removal: After mobilizing the tumor-bearing pelvic segment laterally, the pelvic floor muscles on the ischial side were divided. The sacrotuberous and sacrospinous ligaments were then transected to complete separation and removal of the affected limb.

### 2.4. Postoperative Management

Postoperatively, patients were placed in the supine position, with the affected limb elevated in limb-salvage cases. Prophylactic measures to prevent deep vein thrombosis were routinely implemented. In the absence of significant pain, isometric muscle contraction exercises were initiated shortly after surgery. Approximately two weeks postoperatively, once the incision had healed, the intensity and frequency of rehabilitation training were gradually increased, with knee flexion progressing up to 90 degrees.

For patients who underwent prosthetic joint replacement, hip flexion exercises up to 90 degrees began at three weeks postoperatively, accompanied by partial weight-bearing ambulation using bilateral crutches. At six months, patients were permitted to walk with a single crutch following radiographic confirmation of recovery, and full unassisted ambulation was typically achieved by 12 months. For those who underwent hip arthrodesis, functional training was initiated under brace protection after incision healing, with ambulation using bilateral crutches beginning within the first year and eventually progressing to independent walking.

During the first one to two years after surgery, plain radiographs were obtained every 3 to 6 months. In addition, chest CT, contrast-enhanced pelvic CT, and whole-body bone scans were performed every 6 months. From years 3 to 5 postoperatively, follow-up evaluations were conducted semiannually. Postoperative functional outcomes were assessed using the Musculoskeletal Tumor Society (MSTS) scoring system.

### 2.5. Statistical Analysis

Descriptive statistics were used to analyze the data. Survival was estimated using Kaplan–Meier curves. All analyses were performed using Microsoft Excel (Redmond, Washington, DC, USA) and SPSS statistical software (version 20.0, IBM, Armonk, NY, USA).

## 3. Results

### 3.1. General Results

All patients successfully underwent surgery. The average operative time was 252 min (median: 240 min, range: 120–570 min), with an average intraoperative blood loss of 1358 mL (median: 1200 mL, range: 400–3000 mL). The mean length of hospital stay was 22 days (median: 18 days, range: 12–40 days). All patients completed follow-up, with a mean follow-up duration of 37 months (median: 31 months, range: 14–86 months).

Among them, two patients underwent marginal resection—one involving a Type I + II resection and the other a Type II + III resection—with an average operative time of 367.5 min.

The remaining 17 patients received wide resection, including 4 cases of Type I, 2 cases of Type II, 5 cases of Type III, 2 cases of Type IV, 3 cases of Type II + III, and 1 case of Type I + II. The average operative time in this group was 238.5 min (median: 185 min, range: 120–570 min).

Two patients developed postoperative wound non-healing, which was resolved with surgical debridement.

The statistical analysis of surgical errors has been extensively discussed in our previous work [15], where we further analyzed the modeling of surgical errors and intra-patient variability.

### 3.2. Oncological Results

**Figure 4:**
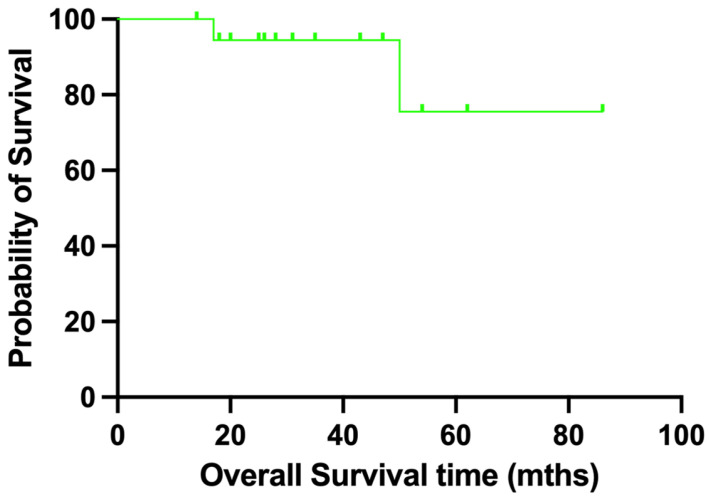
Overall survival curve of 19 patients who underwent pelvic bone tumor resections at our institution using the proposed dangerous region generation method in combination with computer-assisted surgical techniques.

Postoperative pathological assessment confirmed that all cases achieved adequate surgical margins. The five-year overall survival rate was 75.6% (Figure 4). In the group that underwent wide-margin resection, the five-year overall survival rate was 80.0%. During follow-up, two patients died. One patient with sarcoma developed pulmonary metastases 17 months after surgery and died 33 months postoperatively. Another patient with chondrosarcoma developed pulmonary metastases 10 months after surgery and died 50 months postoperatively.

### 3.3. Function Evaluation

At the final postoperative follow-up, MSTS scores were evaluated. Among the 16 surviving patients who underwent limb-salvage surgery, the mean MSTS score was 26.6 (median: 27; range: 21–30), corresponding to an average functional recovery rate of 88.5% (median: 90.0%; range: 70.0–100.0%). The average gait score was 4 (median: 4; range: 3–5). Twelve patients exhibited a normal gait, while four showed mild gait abnormalities. Thirteen patients were able to walk without assistive devices, and three required a single crutch for ambulation (Table 2).

## 4. Discussion

In computer-assisted bone tumor resection surgery, orthopedic surgeons are typically required to preoperatively plan a series of osteotomy planes with sufficient safety margins based on the patient’s imaging data [16,17]. These planes are intended to minimize the risk of residual tumor cells. During the actual operation, the surgeon performs osteotomies with the aid of a computer navigation system, using the preplanned planes as visual references [18]. However, such manual, experience-dependent planning often lacks intuitive feedback on the spatial relationship between the tumor and the planned margins in three-dimensional space. As a result, it is susceptible to errors influenced by subjective judgment or imaging resolution, which may cause discrepancies between the planned osteotomy planes and the ideal resection margins. Inadequate margins not only increase the risk of residual tumor but may also lead to local recurrence or distant metastasis, thereby directly compromising long-term survival and prognosis. To address these challenges, we proposed an efficient three-dimensional dangerous region generation method. This technique uniformly expands the tumor region in three-dimensional space according to a predefined safety margin, rapidly and accurately constructing a complete 3D dangerous region. The generated region delineates both the tumor and its surrounding safety zone, providing a clear and intuitive spatial reference for surgical planning. In practice, surgeons only need to confirm whether the preplanned osteotomy planes lie outside the generated dangerous region to efficiently and reliably design a set of qualified resection planes. This approach substantially reduces reliance on repeated manual estimations, improves both efficiency and accuracy of preoperative planning, and helps minimize the risk of tumor residue or recurrence caused by insufficient margins. The method integrates seamlessly into existing surgical planning workflows, differing primarily in that planning is guided by the dangerous region rather than the tumor region alone. This modification simplifies the definition of cutting planes and typically shortens planning time. Consequently, the expected learning curve for adopting the method is relatively short. Although the current study did not quantitatively assess the learning curve, we have added a discussion noting that future prospective studies will evaluate user experience and inter-surgeon consistency when introducing the method into routine clinical practice. In this study, we conducted clinical follow-up of 19 patients with pelvic bone tumors who underwent surgery assisted by this method. Favorable outcomes were observed: among limb-salvage patients, the mean MSTS functional recovery rate was 88.5%, and no local recurrence was reported during the follow-up period. These findings confirm the feasibility of integrating the dangerous region generation method with computer-assisted pelvic tumor resection, demonstrating its potential to achieve favorable oncological and functional outcomes.

In terms of oncological outcomes, the most critical determinant of recurrence risk following pelvic malignancy resection is the achievement of adequate surgical margins. By uniformly expanding the tumor region in three-dimensional space based on a predefined safety margin, surgeons can compare the preplanned osteotomy planes with the generated dangerous region, thereby rapidly and accurately designing a set of qualified osteotomy planes [19,20]. According to the most recent follow-up results, the local recurrence risk in our cohort was comparable to, or even lower than, that reported in studies of conventional computer-assisted pelvic tumor resections [21,22,23]. During the follow-up period, no local recurrence was observed, and two patients died due to tumor-related pulmonary metastases. As the dangerous region generation method combined with computer-assisted surgery enabled precise osteotomy and complete tumor removal with negative margins, it is likely that these metastatic events resulted from the natural progression of the disease rather than incomplete resection [24]. This finding further supports the efficacy of our technique in achieving safe margins while preserving surrounding healthy bone tissue.

For functional outcomes, among the 16 limb-salvage patients, the mean MSTS score was 26.6 (median: 27; range: 21–30), corresponding to an average functional recovery rate of 88.5% (median: 90.0%; range: 70.0–100.0%). The satisfactory functional outcomes can be attributed to two key factors.

The first factor is detailed preoperative planning. The pelvic region has a highly complex anatomy, closely adjacent to critical functional and vital structures such as the hip joint, sciatic nerve, and iliac vessels [25]. Moreover, the tumor location, morphology, and extent vary considerably among different patients. By applying the dangerous region generation method, individualized osteotomy planes can be designed based on patient-specific anatomy and tumor characteristics, allowing for complete tumor removal while maximally preserving normal bone stock and joint structures, thereby maintaining lower limb function and quality of life to the greatest extent possible [26,27]. And the second factor is the precise execution of the osteotomy plan. In pelvic limb-salvage surgery, the accuracy of osteotomy is directly related to the balance between oncological control and postoperative functional recovery [28,29]. Given the anatomical complexity of the pelvis, where joints, nerves, and blood vessels are densely distributed, excessive resection may result in unnecessary loss of bone and soft tissue, compromise pelvic stability, impair weight-bearing and gait, and even lead to a marked decline in quality of life. Conversely, insufficient resection or positive margins significantly increase the risk of local recurrence and adversely affect long-term survival. Therefore, under the premise of ensuring negative margins and complete tumor removal, maximal preservation of healthy bone can reduce the difficulty of reconstruction, improve the fit and durability of prosthetic or biological reconstructions, and retain more of the native biomechanical support structures [30].

Through accurate preoperative planning, the surgical duration can be effectively reduced. The average operative time was 252 min (median: 240 min; range: 120–570 min), which is lower than that reported in studies on pelvic tumor resection surgery using conventional computer navigation (typically ranging from 333 to 355 min) [17,31,32]. The dangerous region generation method proposed in this study constructs a complete three-dimensional hazardous zone by uniformly expanding the bone tumor region, providing a clear and intuitive spatial reference for preoperative planning. This approach facilitates surgeons in performing intraoperative procedures more efficiently, minimizing unnecessary steps, thereby optimizing operative time and controlling intraoperative blood loss. In this study, the average intraoperative blood loss was 1358 mL (median: 1200 mL; range: 400–3000 mL), which is also lower than the blood loss reported in the studies on pelvic tumor resection with conventional computer navigation [18,21,31]. Several limitations should be acknowledged. First, the study lacked a control group. Second, the small sample size and relatively short follow-up period may introduce bias and limit the generalizability of the findings. Third, the data on soft-tissue margins were not systematically recorded or quantified. In addition, inter-annotator consistency was not quantitatively evaluated in the current dataset. Future work will include multi-annotator assessments to quantify annotation consistency, as well as the establishment of a standardized annotation protocol. These efforts will support the construction of a larger, well-validated bone tumor imaging dataset to facilitate further algorithmic and clinical research.

## 5. Conclusions

We propose an efficient three-dimensional “dangerous region” generation method, which not only accurately delineates the tumor mass and the surrounding safety margins that need to be resected, but also provides a clear and intuitive spatial reference for preoperative planning and osteotomy plane design. The results of this study indicate that this dangerous region generation method, when combined with computer-assisted techniques for pelvic bone tumor resection, is feasible and can achieve favorable clinical outcomes.

## Figures and Tables

**Table 1:** Primary information of 19 patients who underwent pelvic bone tumor resections.

ID	Age	Gender	Tumor Position	Histological Grade	Tumor Type	Follow-Up Time
1	60	male	type IV	IIB	chordoma	86
2	56	male	type II + III	IIB	osteosarcoma	17
3	39	female	type I	IIB	peripheral nerve sheath tumor	62
4	51	female	type II + III	3	giant cell tumor	62
5	66	male	type III	IIB	spindle cell sarcoma	54
6	46	female	type II	IIB	chondrosarcoma	50
7	45	female	type I + II	3	giant cell tumor	47
8	57	female	type I	3	giant cell tumor	43
9	45	female	type III	IB	chondrosarcoma	35
10	40	female	type III	IB	chondrosarcoma	31
11	22	female	type II + III	IIB	Ewing sarcoma	31
12	49	female	type I	3	Chondromyxoid fibroma	28
13	39	female	type III	IB	chondrosarcoma	26
14	57	male	type I + II	IB	chondrosarcoma	25
15	19	male	type I	IIB	Ewing sarcoma	25
16	49	male	type IV	IB	chordoma	20
17	37	male	type III	3	giant cell tumor	20
18	29	female	type II + III	IIB	Ewing sarcoma	18
19	50	male	type II	IB	chondrosarcoma	14

**Table 2:** General results, oncological and functional results of 19 patients bone tumor resection surgeries.

ID	Osteotomy Margin	Operative Time (min)	Intraoperative Blood Loss (mL)	MSTS	Recurrence	Metastasis	Status
1	20 mm	280	1000	21	No	No	Live
2	20 mm	375	2000	30	No	Yes	Dead
3	20 mm	180	800	25	No	No	Live
4	15 mm	300	2400	27	No	No	Live
5	20 mm	240	1000	30	No	No	Live
6	20 mm	160	800	27	No	Yes	Dead
7	10 mm	360	1500	26	No	No	Live
8	10 mm	150	1200	27	No	No	Live
9	20 mm	150	1200	28	No	No	Live
10	20 mm	185	500	27	No	No	Live
11	20 mm	344	2500	26	No	No	Live
12	20 mm	180	400	28	No	No	Live
13	20 mm	240	1400	27	No	No	Live
14	20 mm	180	400	-	No	No	Live
15	20 mm	570	3000	28	No	No	Live
16	15 mm	165	1200	27	No	No	Live
17	10 mm	120	1000	27	No	No	Live
18	20 mm	340	1000	25	No	No	Live
19	20 mm	270	2500	26	No	No	Live

## Data Availability

The data presented in this study are available upon request from the corresponding author.
